# Editorial: Multi-omics application in exploring potential biomarkers targeting resistance of anti-cancer drugs

**DOI:** 10.3389/fphar.2025.1594064

**Published:** 2025-04-16

**Authors:** Huan Wang, Jindong Xie, Zhi Tian, Diane S. Allen-Gipson, Hailin Tang

**Affiliations:** ^1^ State Key Laboratory of Oncology in South China, Guangdong Provincial Clinical Research Center for Cancer, Sun Yat-sen University Cancer Center, Guangzhou, China; ^2^ Department of Molecular Pharmacology and Physiology, Morsani College of Medicine, Unversity of South Florida, Tampa, FL, United States; ^3^ USF Health Taneja College of Pharmacy, Department of Pharmaceutical Sciences, University of South Florida, Tampa, FL, United States

**Keywords:** multi-omics, biomarker, drug resistance, oncology, tumor microenvironment

Anti-cancer drug resistance denotes the capacity of cancer cells to withstand the effects of therapeutic agents. This phenomenon is both highly prevalent and complex within the context of cancer treatment, and it constitutes a major factor contributing to diminished therapeutic efficacy and adverse patient outcomes (Gao et al., 2024). Cancer drug resistance constitutes a fundamental challenge in contemporary oncology, stemming from the intricate regulation of complex biological networks by tumor cells to circumvent drug-induced cytotoxic effects (Lei et al., 2020). To systematically elucidate this complex mechanism, multi-omics technologies have emerged as a pivotal advancement in contemporary research. Through the integration of multidimensional data derived from genomics, transcriptomics, proteomics, metabolomics, and additional omics layers, multi-omics studies develop a comprehensive atlas of tumor biological systems (Kreitmaier et al., 2023). In comparison to traditional single-omics analyses, this integrative approach effectively captures cascade regulatory relationships across molecular hierarchies, thereby elucidating the network-based mechanisms that underlie drug resistance. In the field of biomarker discovery, multi-omics approaches offer distinct advantages. In the realm of biomarker discovery, multi-omics technologies demonstrate distinct advantages. For instance, through the integration of transcriptomic and proteomic approaches, researchers can elucidate how neoplastic cells evade pharmacological interventions by modifying gene expression profiles and altering protein functional states (Xie et al., 2020). Regarding the investigation of drug resistance mechanisms, multi-omics methodologies equip researchers with robust analytical tools. The systematic integration of metabolomic datasets with systems biology modeling enables comprehensive delineation of molecular pathways underlying therapeutic resistance (Eicher et al., 2020). The current Research Topic, “Multi-omics Application in Exploring Potential Biomarkers Targeting Resistance of Anti-Cancer Drugs”, convenes leading researchers in this highly anticipated field to present a Research Topic of authoritative reviews and compelling original articles. These articles provide an in-depth understanding and an innovative, comprehensive perspective on drug resistance mechanisms, multi-omics methodologies, and potential strategies for overcoming drug resistance.

Advancements in multi-omics technologies, particularly those achieving single-cell resolution and spatiotemporal dynamic analysis, are increasingly elucidating the complex regulatory mechanisms underlying cancer drug resistance. These developments present new opportunities for the formulation of innovative therapeutic strategies. Pharmaco-omics has emerged as a prominent research frontier. Wu et al. conducted integrative analyses combining pharmaco-omics with genomic and transcriptomic datasets, that elevated expression of CLDN18.2 is significantly associated with poor prognosis in bladder cancer (BLCA), esophageal carcinoma (ESCA), and pancreatic adenocarcinoma (PAAD). This study comprehensively elucidates the biological functions and clinical relevance of CLDN18.2 in cancer, thereby offering novel insights for the development of targeted therapies. Similarly, He et al. employed multidimensional integration of pharmaco-omics, genomics, and immunomics to characterize the landscape of extrachromosomal circular DNA (eccDNA) in prostate adenocarcinoma (PRAD). Their research identified eccDNA-derived ZNF330 and PITPNM3 as potential biomarkers. Their risk stratification model provides novel insights for prognostic assessment and the development of immunotherapy strategies. Expanding methodological approaches, Huang et al. further integrated pharmaco-omics with epigenetic profiling and single-cell omics to investigate the pan-cancer expression patterns, prognostic significance, and associations of MCM3 with tumor immune microenvironments, subsequently validating its prognostic utility in a clinical cohort of lower-grade glioma (LGG).

In addition to pharmaco-omics, proteomic investigations remain a prominent focus in oncology research. Peng et al. employed proteomics, genomics, and bioinformatics tools to explore the function of indoleamine 2,3-dioxygenase 1 (IDO1) within the tumor microenvironment (TME) of esophageal squamous cell carcinoma (ESCC). Their findings indicate that tumor-associated macrophages (TAMs) with elevated IDO1 expression contribute to an immunosuppressive TME, thereby reducing the effectiveness of immunotherapy. Through an analysis of RNA-seq data from The Cancer Genome Atlas (TCGA) involving 95 patients, supplemented by clinical validation in 77 patients, they found that targeting IDO1 in TAMs could serve as a viable strategy to counteract immune resistance. This underscores the potential of IDO1 inhibitors as adjunctive agents to improve the efficacy of immunotherapeutic interventions. In parallel, Huang et al. conducted a multidimensional analysis by integrating proteomics, transcriptomics, and clinical omics to explore the expression patterns, prognostic value, immune signatures, and clinical relevance of the minichromosomal maintenance complex component 3 (MCM3) gene across pan-cancer cohorts, with specific validation in lower-grade glioma (LGG). Their work comprehensively elucidated the molecular mechanisms and clinical potential of MCM3 in oncology.

Zhang et al. combined transcriptomic and proteomic data to explore the role of mitochondrial PCK2 in NSCLC. They found that PCK2-driven gluconeogenesis helps cancer cells evade mitochondrial apoptosis, indicating that targeting metabolic pathways like gluconeogenesis could be a strategy to combat drug resistance in nutrient-poor tumor environments. Wang et al. further advanced this field by combining proteomics and metabolomics to study hepatocellular carcinoma (HCC) resistance mechanisms to tyrosine kinase inhibitors (TKIs) sorafenib and lenvatinib, providing a global perspective on HCC drug resistance and facilitating the discovery of novel therapeutic targets.

The integration of genomics and transcriptomics remains a prominent research direction. Su et al. combined genomics (IRF1 mutation analysis, target gene prediction), transcriptomics (GSEA pathway enrichment, correlations between gene expression and immune factors), and single-cell omics (single-cell transcriptomic profiling of IRF1 distribution) to investigate the clinical significance and biological functions of interferon regulatory factor 1 (IRF1) in NSCLC patients undergoing chemoimmunotherapy. Similarly, Xu et al. integrated multidimensional data (gene expression, mutations, immune cell infiltration, clinical outcomes) to analyze associations between pyroptosis-related genes (PRGs) and the tumor immune microenvironment (TME), constructing a predictive model for immunotherapy response and prognosis. Li et al. leveraged transcriptomics (RNA sequencing), genomics (somatic mutation profiling), and single-cell RNA sequencing (scRNA-seq) to evaluate the predictive value of epithelial-mesenchymal transition (EMT)-related genes in osteosarcoma (OS) prognosis, immune infiltration, and therapeutic response.

Additional exploratory studies, while not employing multi-omics technologies, have innovatively proposed novel strategies to address anti-cancer drug resistance. Shen et al. investigated the use of PEG-PLGA nanoparticles loaded with berberine (Ber) to enhance inhibitory effects on colorectal cancer (CRC). Utilizing transcriptomics (RNA-Seq), they analyzed gene expression changes in HCT116 cells treated with free Ber *versus* nanoparticle-encapsulated Ber (NPBer), clarifying its regulatory effects on critical signaling pathways and biological processes. Chen et al. conducted targeted mechanistic studies on flavonoid derivatives (DMF: 4′,5-dihydroxy-7-piperazinylmethoxy-8-methoxyflavone) extracted and chemically modified from the traditional Chinese herb Sorbaria sorbifolia, elucidating their antitumor effects on hepatocellular carcinoma (HCC) cells and underlying molecular mechanisms. Li et al. explored the role of dorsomorphin in suppressing ABCG2-mediated multidrug resistance (MDR) in CRC. They demonstrated that dorsomorphin reverses MDR by directly inhibiting ATP-binding cassette transporters2 (ABCG2) transporter activity, identifying it as a potential multi-target inhibitor, though further validation of its *in vivo* efficacy is required. Yang et al. compared the efficacy and safety of two chemotherapy regimens (NP group: nedaplatin + liposomal paclitaxel vs. ND group: nedaplatin + docetaxel) in platinum-sensitive recurrent ovarian cancer (ROC) patients. By retrospectively analyzing clinical data (e.g., FIGO staging, number of recurrent lesions) from 121 patients, they found that the ND regimen conferred superior survival benefits with manageable toxicity, supporting personalized therapeutic strategies.

The thematic scope also includes review articles focusing on high-impact research directions. For instance, Song et al. systematically summarized the molecular mechanisms of cell cycle regulation, the principles of targeted therapies, and preclinical/clinical trial data, highlighting the application of cell cycle checkpoints and their inhibitors in cancer treatment. Wang et al. comprehensively reviewed the roles of long non-coding RNAs (lncRNAs) in urological cancers (prostate, bladder, renal cancers), emphasizing their regulation of autophagy in cancer progression, therapy resistance, and biomarker potential, with partial integration of transcriptomic and epigenetic omics technologies. Chen’s article focused on statistical meta-analyses of clinical data, systematically evaluating the survival impacts of various drugs (e.g., metformin, statins, β-blockers, aspirin) in gynecologic malignancies (ovarian, endometrial cancers). Tang et al. reviewed the central role of SIRT1 (Sirtuin 1) in cancer autophagy and drug resistance, emphasizing molecular mechanisms, signaling pathways, and preclinical models (e.g., gene knockout, inhibitor treatment, cell/animal studies).

In summary, therapeutic resistance to antineoplastic agents remains a formidable challenge in oncology. Addressing cancer drug resistance necessitates the development of innovative therapeutic strategies, including immunotherapy, gene editing, and precision medicine paradigms, as well as collaborative efforts across multiple disciplines spanning basic research, clinical investigations, and public health initiatives. Chemoresistance represents a central obstacle in cancer management, and its investigation not only facilitates improved patient prognostication but also establishes critical directions for future therapeutic innovation. Key research priorities include mechanistic investigations of resistance, novel drug development, combination therapeutic regimens, and individualized treatment approaches. The integrative application of multi-omics technologies provides transformative conceptual frameworks for these endeavors, enabling the identification of novel biomarkers and therapeutic targets. Such advancements hold potential to enhance therapeutic efficacy, circumvent resistance mechanisms, and generate promising pathways for translational research and clinical implementation. It is our expectation that discoveries in this field will catalyze further innovation, with anticipation of groundbreaking progress in the evolving landscape of oncology therapeutics.
